# Editorial: Prevention in Acute Leukemias in Children

**DOI:** 10.3389/fpubh.2021.700739

**Published:** 2021-08-09

**Authors:** Juan M. Mejia-Arangure, Richard J. Q. McNally, Maria S. Pombo-de-Oliveira

**Affiliations:** ^1^Coordinación de Investigación en Salud, Instituto Mexicano del Seguro Social, Mexico & Facultad de Medicina, Universidad Nacional Autónoma de México, Mexico City, Mexico; ^2^Newcastle University, Newcastle upon Tyne, United Kingdom; ^3^Research Center, Instituto Nacional de Câncer, Rio de Janeiro, Brazil

**Keywords:** acute lymphoblastic leukemia, epidemiology–analytic (risk factors), children, cancer in children and adolescents, etiology, prevention

The types of acute leukemia (AL) in children are heterogeneous diseases with different etiology, and, therefore, their prevention may be diverse and complex. Improved prevention not only depends on identifying the relevant risk factors associated with the types of AL but also on identifying the patients who are at higher risk of developing the disease and in whom the prevention measures could be more useful.

The incidence rate of the types of AL varies among countries around the world. Acute lymphoblastic leukemia (ALL) is more frequent in Caucasian and Hispanic populations (1). There are reported differences with regard to peak incidence, immunophenotype, and cytogenetic subtypes, which vary by geographical regions and by populations (2).

During recent years, the incidence of the types of AL, especially B cell ALL, has risen worldwide, but in Latin America, this increase has been much higher (3, 4). Plausible hypotheses include the role of jobs with lower standards of control to carcinogenic exposures, migration to countries with worse conditions concerning environmental exposures at work, and situations of unusual population mixing could increase the risk of ALL, which is described by Kinlen's hypothesis (2).

Changes in the environment and the presence of pandemic agents could affect the incidence of the types of AL, especially of ALL (5, 6). For instance, the rate of cesarean sections (C-S) as a mode of delivery has significantly increased over time in several countries (from 18.6 to 55.9%) (7, 8), in parallel with increased prevalence of chronic immune disorders in childhood including ALL (9). Therefore, C-S exemplifies an environmental factor that allows prevention with policies to avoid unnecessary surgery in preterm births. Early exposure to viral infections could reduce the risk of ALL, and in recent years, it has been postulated that the microbiome could be associated with this increased risk (5).

It is clear that the etiology of the types of AL depends on genetic susceptibility, including constitutional susceptibility (e.g., Down syndrome), and is also related to carcinogenic metabolism of immunity to different infectious agents (2). The age peak of the incidence of ALL and the acute myeloid leukemias (AML), among 2 and 5 years, and during the first year, respectively, might be the result of a window of susceptibility where the environmental exposures would be more important in the induction of the development of AL; however, in Africa and Latino American countries, the high incidence in adolescents with respect to other countries is another point to consider (10). To develop any type of AL, it is necessary to be exposed to carcinogenic agents, some agents could induce cancer, but others could promote this cancer, such as the electromagnetic fields (2) or infections, such as viruses.

In this supplement, we have collected principal manuscripts, where susceptibility of the types of AL and the interaction between susceptibility and the exposures was assessed, and, also, the important viewpoints about screening and the implication of the differences of subtypes of AML in the incidence and the response to the treatment were analyzed.

To discuss the prevention of the types of AL is not so common, but this topic should be further researched by the new generation of researchers. The new research on the types of AL must consider this possibility. Many years of work have led to a better understanding of the etiology of the types of AL, but now the focus must be on the perspective of improved prevention.

We cannot prevent any type of AL with the identification of only one factor capable of causing AL, but we need to take the first step toward its prevention. We need to focus on the prevention of the different types of leukemia. We have observed some putative factors, such as parental smoking or alcohol consumption, before the time of conception, but we need to investigate those from the perspective of prevention. We need to make decisions and implement strategies in populations where the incidence of the disease is higher, the presentation of the disease is worst, and where the health system has a poor record of survival of these patients.

We want this supplement to be a step toward creating awareness for the prevention of leukemia.

## Author Contributions

All authors listed have made a substantial, direct and intellectual contribution to the work, and approved it for publication.

## Conflict of Interest

The authors declare that the research was conducted in the absence of any commercial or financial relationships that could be construed as a potential conflict of interest.

## Publisher's Note

All claims expressed in this article are solely those of the authors and do not necessarily represent those of their affiliated organizations, or those of the publisher, the editors and the reviewers. Any product that may be evaluated in this article, or claim that may be made by its manufacturer, is not guaranteed or endorsed by the publisher.
